# Complete Genome Sequences of Two Novel Species from the *Pseudonocardiaceae* Family Isolated from the Persian Gulf

**DOI:** 10.1128/MRA.00918-21

**Published:** 2021-12-16

**Authors:** Nasim Safaei, Boyke Bunk, Cathrin Spröer, Joachim Wink, Yvonne Mast

**Affiliations:** a Department of Microbial Strain Collection, Helmholtz Centre for Infection Research, Braunschweig, Germany; b Leibniz Institute, German Collection of Microorganisms and Cell Cultures, Braunschweig, Germany; University of Southern California

## Abstract

*Amycolatopsis* sp. strain DSM 110486 and *Pseudonocardia* sp. strain DSM 110487 are two novel actinomycete species that were isolated from Hengam Island beach sand from the Persian Gulf. Here, we present the complete genome sequences of DSM 110486 and DSM 110487, with sizes of 10.98 Mbp and 10.33 Mbp, respectively.

## ANNOUNCEMENT

The family *Pseudonocardiaceae* includes several well-known natural-compound-producing genera, including *Amycolatopsis* and *Pseudonocardia*. In the context of a screening approach for novel natural compound producers, two new actinomycete strains (DSM 110486 and DSM 110487) have been isolated from a sand sample from Hengam Island, following the isolation method described by Safaei et al. (1). Hengam Island is a small island (37 km**^2^**) located south of Qeshm Island in the Persian Gulf (26.674191, 55.904151).

For DNA isolation, strains DSM 110486 and DSM 110487 were grown in glucose-yeast-malt (GYM) medium for 14 days at 30°C on a rotary shaker (160 rpm). DNA was isolated using a Genomic-tip 100/G (Qiagen, Hilden, Germany) according to the instructions of the manufacturer and was used for the preparation of long and short-read libraries. A SMRTbell template library for sequencing on the Sequel II platform was prepared according to the instructions from Pacific Biosciences (PacBio) (Menlo Park, CA, USA), following the procedure and checklist for preparing multiplexed microbial libraries using SMRTbell Express template preparation kit v2.0. Libraries for sequencing on the NextSeq 500 platform were prepared using the Nextera XT DNA library preparation kit (Illumina, San Diego, CA, USA) with modifications according to Baym et al. (2). Preassembly read quality control (QC) was performed using single-molecule real-time (SMRT) Link (Run QC module) and FastQC (https://www.bioinformatics.babraham.ac.uk/projects/fastqc). Long-read genome assembly was performed using the Microbial Assembly protocol included in SMRT Link v10.1.0, applying target genome sizes of 10.9 and 10.3 Mbp for DSM 110486 and DSM 110487, respectively. The assembly process resulted in one circular chromosomal contig and one linear plasmid each. Genome completeness was retrieved from the Microbial Assembly results in terms of circular replicons or blunt-end linear replicons, as well as 0% missing bases. Error correction was performed by mapping of Illumina short reads onto the completed genome using BWA (3), with subsequent determination of a new consensus sequence (https://github.com/JHartlich/AlternateReferenceMaker). Genome annotation was performed with the NCBI Prokaryotic Genome Annotation Pipeline (PGAP). Default parameters were used for all software. All genome-sequence-related data are summarized in Table 1.

**TABLE 1:** Sequencing and annotation data

Parameter	Data for:	
	DSM 110486	DSM 110487
Genome length (bp)	10,984,156	10,329,491
No. of chromosomes	1	1
No. of extrachromosomal elements	1	1
Genome coverage (×)	533	343
Avg G+C content (%)	70.2	71.8
No. of coding sequences	10,508	9,666
No. of tRNAs	70	47
No. of rRNAs	12	6
No. of PacBio reads	156,372	88,199
PacBio read *N*_50_ (bp)	40,948	65,481
No. of Illumina reads	3,002,725	4,701,445
Illumina paired-end read length (bp)	2 × 151	2 × 151
GenBank accession no.		
Chromosome	CP080519.1	CP080521.1
Plasmid	CP080520.1	CP080522.1
BioProject no.	PRJNA751440	PRJNA751442
SRA accession no.		
PacBio reads	SRX12487219	SRX12487266
Illumina reads	SRX12487220	SRX12487267

**FIG 1:**
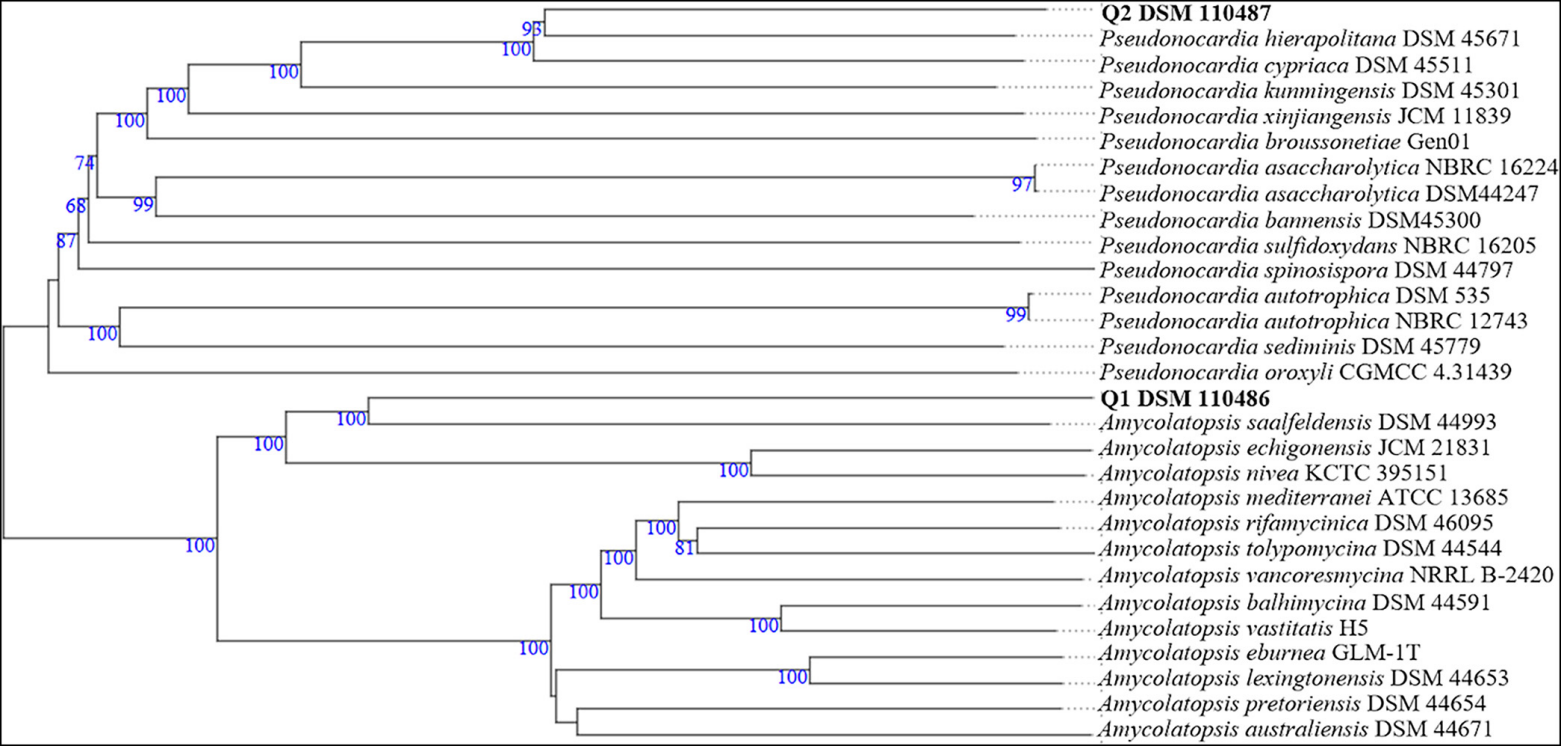
Whole-genome sequence tree generated with the TYGS web server for strains DSM 110486 and DSM 110487 and closely related species. The tree was inferred with FastME from Genome BLAST Distance Phylogeny (GBDP) distances calculated from the genome sequences. The branch lengths are scaled in terms of GBDP distance formula *d_5_*. The numbers above the branches are GBDP pseudo-bootstrap support values of >60% from 100 replications, with an average branch support value of 90.3%. The tree was rooted at the midpoint.

Phylogenomic analysis of the full-length genome sequences with the Type (Strain) Genome Server (TYGS) v1.0 (https://tygs.dsmz.de) (4) revealed that DSM 110486 and DSM 110487 are most closely related to the type strains Amycolatopsis saalfeldensis DSM 44993 and Pseudonocardia hierapolitana DSM 45671 (Fig. 1), with digital DNA-DNA hybridization (dDDH) values (formula *d_4_*) of 27.2% and 36.4%, respectively, suggesting that both strains represent novel species. AntiSMASH v6.0 analysis (5) with the genome sequences of DSM 110486 and DSM 110487 led to the identification of 23 and 16 biosynthetic gene clusters, respectively, revealing the genetic potential of these strains for the production of novel natural products.

### Data availability.

Genome sequence-related data availability is listed in Table 1.
